# Supplementary material for: Time-series transcriptome analysis identified differentially expressed genes in broiler chicken infected with mixed Eimeria species
Source: Front Genet. 2022 Aug 8;13:886781. doi: 10.3389/fgene.2022.886781 (PMC9393255; doi:10.3389/fgene.2022.886781)
Supplement: Supplementary file 2 [file DataSheet1.ZIP › 7dpi_KEGG.Gsea.1625115961950/KEGG_PATHWAYS_IN_CANCER.html]

Details for gene set KEGG\_PATHWAYS\_IN\_CANCER[GSEA]

|  || Dataset | TMM\_7dpi\_gct\_format\_7dpi\_gct\_format.Class\_4dpi.cls #PC\_versus\_NC.Class\_4dpi.cls #PC\_versus\_NC\_repos |
| Phenotype | Class\_4dpi.cls#PC\_versus\_NC\_repos |
| Upregulated in class | 0 |
| GeneSet | KEGG\_PATHWAYS\_IN\_CANCER |
| Enrichment Score (ES) | -0.3961206 |
| Normalized Enrichment Score (NES) | -1.522286 |
| Nominal p-value | 2.0104543E-4 |
| FDR q-value | 0.12074389 |
| FWER p-Value | 0.9326 |
Table: GSEA Results Summary

  

Fig 1: Enrichment plot: KEGG\_PATHWAYS\_IN\_CANCER      
 Profile of the Running ES Score & Positions of GeneSet Members on the Rank Ordered List

  

| SYMBOL | TITLE | RANK IN GENE LIST | RANK METRIC SCORE | RUNNING ES | CORE ENRICHMENT || 1 | PPARG | na | 83 | 1.306 | 0.0028 | No |
| 2 | STAT1 | na | 103 | 1.240 | 0.0106 | No |
| 3 | BMP2 | na | 134 | 1.164 | 0.0169 | No |
| 4 | SLC2A1 | na | 178 | 1.078 | 0.0214 | No |
| 5 | PPARD | na | 309 | 0.931 | 0.0174 | No |
| 6 | CYCS | na | 475 | 0.763 | 0.0092 | No |
| 7 | CASP8 | na | 514 | 0.732 | 0.0115 | No |
| 8 | BCL2L1 | na | 568 | 0.700 | 0.0123 | No |
| 9 | NOS2 | na | 593 | 0.688 | 0.0155 | No |
| 10 | RBX1 | na | 729 | 0.622 | 0.0087 | No |
| 11 | RARA | na | 749 | 0.611 | 0.0117 | No |
| 12 | CDKN1A | na | 843 | 0.575 | 0.0082 | No |
| 13 | HDAC1 | na | 908 | 0.552 | 0.0069 | No |
| 14 | FH | na | 1026 | 0.512 | 0.0009 | No |
| 15 | PGF | na | 1105 | 0.483 | -0.0021 | No |
| 16 | WNT1 | na | 1130 | 0.474 | -0.0006 | No |
| 17 | FGF7 | na | 1167 | 0.467 | -0.0001 | No |
| 18 | RASSF1 | na | 1321 | 0.424 | -0.0099 | No |
| 19 | HRAS | na | 1342 | 0.420 | -0.0084 | No |
| 20 | MAX | na | 1375 | 0.411 | -0.0080 | No |
| 21 | MECOM | na | 1573 | 0.361 | -0.0220 | No |
| 22 | BID | na | 1694 | 0.334 | -0.0297 | No |
| 23 | BMP4 | na | 1723 | 0.327 | -0.0296 | No |
| 24 | LAMB4 | na | 1746 | 0.321 | -0.0290 | No |
| 25 | WNT5B | na | 1855 | 0.297 | -0.0359 | No |
| 26 | TCF7L1 | na | 1896 | 0.288 | -0.0372 | No |
| 27 | TGFA | na | 1901 | 0.287 | -0.0353 | No |
| 28 | TGFB2 | na | 1954 | 0.278 | -0.0376 | No |
| 29 | SUFU | na | 1994 | 0.272 | -0.0389 | No |
| 30 | ITGA3 | na | 2012 | 0.270 | -0.0383 | No |
| 31 | CASP9 | na | 2022 | 0.268 | -0.0370 | No |
| 32 | FZD8 | na | 2052 | 0.262 | -0.0375 | No |
| 33 | BCL2 | na | 2059 | 0.261 | -0.0360 | No |
| 34 | AR | na | 2089 | 0.255 | -0.0366 | No |
| 35 | DAPK3 | na | 2102 | 0.253 | -0.0357 | No |
| 36 | FZD7 | na | 2145 | 0.245 | -0.0374 | No |
| 37 | ARNT2 | na | 2323 | 0.218 | -0.0508 | No |
| 38 | TRAF4 | na | 2490 | 0.193 | -0.0634 | No |
| 39 | NFKBIA | na | 2532 | 0.187 | -0.0655 | No |
| 40 | RARB | na | 2792 | 0.151 | -0.0864 | No |
| 41 | ITGA2B | na | 2940 | 0.134 | -0.0978 | No |
| 42 | WNT5A | na | 2943 | 0.133 | -0.0970 | No |
| 43 | BIRC5 | na | 2944 | 0.133 | -0.0960 | No |
| 44 | GLI1 | na | 2982 | 0.129 | -0.0982 | No |
| 45 | RAC1 | na | 2998 | 0.126 | -0.0985 | No |
| 46 | DAPK2 | na | 3012 | 0.125 | -0.0986 | No |
| 47 | MET | na | 3037 | 0.122 | -0.0998 | No |
| 48 | LAMB2 | na | 3242 | 0.094 | -0.1164 | No |
| 49 | CKS1B | na | 3323 | 0.084 | -0.1226 | No |
| 50 | RALA | na | 3386 | 0.075 | -0.1273 | No |
| 51 | VHL | na | 3423 | 0.071 | -0.1298 | No |
| 52 | MAP2K2 | na | 3435 | 0.070 | -0.1302 | No |
| 53 | FGF12 | na | 3438 | 0.069 | -0.1298 | No |
| 54 | RHOA | na | 3449 | 0.068 | -0.1302 | No |
| 55 | TPM3 | na | 3456 | 0.068 | -0.1302 | No |
| 56 | WNT2B | na | 3560 | 0.057 | -0.1385 | No |
| 57 | SMO | na | 3875 | 0.019 | -0.1650 | No |
| 58 | CDK2 | na | 3911 | 0.015 | -0.1679 | No |
| 59 | ERBB2 | na | 3936 | 0.011 | -0.1698 | No |
| 60 | FZD2 | na | 3965 | 0.009 | -0.1722 | No |
| 61 | FGFR1 | na | 4080 | -0.004 | -0.1818 | No |
| 62 | RALB | na | 4141 | -0.011 | -0.1868 | No |
| 63 | EGLN1 | na | 4230 | -0.022 | -0.1942 | No |
| 64 | BIRC3 | na | 4236 | -0.023 | -0.1944 | No |
| 65 | HSP90AB1 | na | 4247 | -0.024 | -0.1951 | No |
| 66 | PDGFB | na | 4266 | -0.026 | -0.1964 | No |
| 67 | PDGFA | na | 4308 | -0.031 | -0.1997 | No |
| 68 | FZD4 | na | 4386 | -0.039 | -0.2059 | No |
| 69 | JUN | na | 4404 | -0.040 | -0.2071 | No |
| 70 | LAMA4 | na | 4435 | -0.043 | -0.2093 | No |
| 71 | FZD1 | na | 4450 | -0.045 | -0.2101 | No |
| 72 | DVL2 | na | 4531 | -0.054 | -0.2165 | No |
| 73 | NFKB2 | na | 4877 | -0.090 | -0.2452 | No |
| 74 | HDAC2 | na | 4971 | -0.100 | -0.2523 | No |
| 75 | VEGFC | na | 5000 | -0.103 | -0.2539 | No |
| 76 | HIF1A | na | 5064 | -0.109 | -0.2584 | No |
| 77 | TRAF1 | na | 5114 | -0.114 | -0.2617 | No |
| 78 | CCDC6 | na | 5125 | -0.115 | -0.2617 | No |
| 79 | RAF1 | na | 5142 | -0.116 | -0.2622 | No |
| 80 | MAP2K1 | na | 5188 | -0.120 | -0.2651 | No |
| 81 | CREBBP | na | 5240 | -0.125 | -0.2685 | No |
| 82 | CCNE2 | na | 5260 | -0.127 | -0.2691 | No |
| 83 | AXIN2 | na | 5268 | -0.128 | -0.2688 | No |
| 84 | FZD6 | na | 5278 | -0.129 | -0.2686 | No |
| 85 | TGFB1 | na | 5297 | -0.130 | -0.2691 | No |
| 86 | LAMB1 | na | 5338 | -0.134 | -0.2715 | No |
| 87 | FOS | na | 5340 | -0.134 | -0.2706 | No |
| 88 | RUNX1T1 | na | 5557 | -0.154 | -0.2878 | No |
| 89 | RUNX1 | na | 5559 | -0.154 | -0.2867 | No |
| 90 | CDKN1B | na | 5564 | -0.155 | -0.2858 | No |
| 91 | PTCH2 | na | 5603 | -0.159 | -0.2879 | No |
| 92 | RALBP1 | na | 5695 | -0.169 | -0.2943 | No |
| 93 | STAT5A | na | 5791 | -0.178 | -0.3010 | No |
| 94 | CDC42 | na | 5837 | -0.184 | -0.3035 | No |
| 95 | RAC3 | na | 6076 | -0.210 | -0.3221 | No |
| 96 | TGFBR2 | na | 6133 | -0.218 | -0.3252 | No |
| 97 | TFG | na | 6152 | -0.220 | -0.3251 | No |
| 98 | FOXO1 | na | 6192 | -0.224 | -0.3267 | No |
| 99 | ARNT | na | 6240 | -0.228 | -0.3290 | No |
| 100 | PLCG1 | na | 6241 | -0.228 | -0.3272 | No |
| 101 | RELA | na | 6283 | -0.233 | -0.3290 | No |
| 102 | CTNNA1 | na | 6367 | -0.240 | -0.3342 | No |
| 103 | FGFR2 | na | 6390 | -0.242 | -0.3342 | No |
| 104 | AXIN1 | na | 6400 | -0.243 | -0.3332 | No |
| 105 | CASP3 | na | 6448 | -0.248 | -0.3353 | No |
| 106 | MAPK8 | na | 6464 | -0.250 | -0.3346 | No |
| 107 | ABL1 | na | 6549 | -0.259 | -0.3398 | No |
| 108 | MLH1 | na | 6647 | -0.270 | -0.3460 | No |
| 109 | CDH1 | na | 6672 | -0.272 | -0.3460 | No |
| 110 | GRB2 | na | 6686 | -0.274 | -0.3450 | No |
| 111 | FZD9 | na | 6854 | -0.292 | -0.3570 | No |
| 112 | CRK | na | 6900 | -0.297 | -0.3586 | No |
| 113 | GLI2 | na | 6927 | -0.300 | -0.3585 | No |
| 114 | PDGFRA | na | 6935 | -0.300 | -0.3569 | No |
| 115 | FASLG | na | 6950 | -0.302 | -0.3557 | No |
| 116 | MYC | na | 6951 | -0.302 | -0.3535 | No |
| 117 | PTGS2 | na | 6986 | -0.306 | -0.3540 | No |
| 118 | TCF7L2 | na | 7019 | -0.311 | -0.3544 | No |
| 119 | MITF | na | 7022 | -0.311 | -0.3522 | No |
| 120 | CDKN2B | na | 7023 | -0.311 | -0.3498 | No |
| 121 | AKT3 | na | 7033 | -0.312 | -0.3482 | No |
| 122 | CRKL | na | 7144 | -0.324 | -0.3551 | No |
| 123 | MMP2 | na | 7168 | -0.325 | -0.3546 | No |
| 124 | E2F1 | na | 7190 | -0.328 | -0.3539 | No |
| 125 | KRAS | na | 7206 | -0.330 | -0.3527 | No |
| 126 | ITGB1 | na | 7209 | -0.331 | -0.3504 | No |
| 127 | MSH2 | na | 7352 | -0.347 | -0.3598 | No |
| 128 | TCF7 | na | 7360 | -0.347 | -0.3578 | No |
| 129 | RXRA | na | 7364 | -0.348 | -0.3554 | No |
| 130 | PIAS4 | na | 7394 | -0.351 | -0.3552 | No |
| 131 | FGF9 | na | 7401 | -0.352 | -0.3530 | No |
| 132 | HGF | na | 7414 | -0.353 | -0.3514 | No |
| 133 | CUL2 | na | 7430 | -0.354 | -0.3500 | No |
| 134 | NRAS | na | 7474 | -0.358 | -0.3509 | No |
| 135 | TGFBR1 | na | 7506 | -0.362 | -0.3508 | No |
| 136 | CTBP2 | na | 7557 | -0.367 | -0.3523 | No |
| 137 | MAPK9 | na | 7648 | -0.377 | -0.3571 | No |
| 138 | CDK6 | na | 7686 | -0.382 | -0.3573 | No |
| 139 | WNT4 | na | 7741 | -0.389 | -0.3590 | No |
| 140 | EP300 | na | 7828 | -0.398 | -0.3632 | No |
| 141 | CCNE1 | na | 7866 | -0.403 | -0.3633 | No |
| 142 | RAC2 | na | 7904 | -0.409 | -0.3634 | No |
| 143 | CTNNA3 | na | 8161 | -0.441 | -0.3818 | No |
| 144 | RB1 | na | 8176 | -0.443 | -0.3796 | No |
| 145 | NCOA4 | na | 8230 | -0.450 | -0.3807 | No |
| 146 | AKT1 | na | 8399 | -0.472 | -0.3914 | No |
| 147 | SHH | na | 8447 | -0.477 | -0.3918 | No |
| 148 | IKBKB | na | 8465 | -0.479 | -0.3896 | No |
| 149 | PIK3CG | na | 8478 | -0.481 | -0.3870 | No |
| 150 | BCR | na | 8519 | -0.485 | -0.3867 | No |
| 151 | SPI1 | na | 8617 | -0.502 | -0.3912 | No |
| 152 | PTEN | na | 8620 | -0.503 | -0.3875 | No |
| 153 | PRKCA | na | 8704 | -0.513 | -0.3907 | No |
| 154 | PDGFRB | na | 8726 | -0.517 | -0.3886 | No |
| 155 | VEGFA | na | 8816 | -0.526 | -0.3921 | Yes |
| 156 | FN1 | na | 8826 | -0.526 | -0.3889 | Yes |
| 157 | JUP | na | 8854 | -0.531 | -0.3872 | Yes |
| 158 | LAMC1 | na | 8856 | -0.531 | -0.3832 | Yes |
| 159 | SOS1 | na | 8869 | -0.533 | -0.3802 | Yes |
| 160 | CSF1R | na | 8919 | -0.538 | -0.3803 | Yes |
| 161 | PLD1 | na | 8979 | -0.546 | -0.3812 | Yes |
| 162 | CSF2RA | na | 8987 | -0.547 | -0.3776 | Yes |
| 163 | CSF3R | na | 9044 | -0.555 | -0.3782 | Yes |
| 164 | PTK2 | na | 9133 | -0.570 | -0.3813 | Yes |
| 165 | CTNNB1 | na | 9201 | -0.583 | -0.3826 | Yes |
| 166 | FZD3 | na | 9213 | -0.585 | -0.3791 | Yes |
| 167 | RAD51 | na | 9288 | -0.597 | -0.3809 | Yes |
| 168 | NFKB1 | na | 9346 | -0.606 | -0.3811 | Yes |
| 169 | EGLN3 | na | 9403 | -0.615 | -0.3812 | Yes |
| 170 | CTBP1 | na | 9420 | -0.618 | -0.3779 | Yes |
| 171 | HSP90B1 | na | 9448 | -0.623 | -0.3755 | Yes |
| 172 | FGFR3 | na | 9457 | -0.624 | -0.3714 | Yes |
| 173 | VEGFD | na | 9472 | -0.627 | -0.3679 | Yes |
| 174 | SKP2 | na | 9511 | -0.633 | -0.3663 | Yes |
| 175 | CHUK | na | 9554 | -0.639 | -0.3650 | Yes |
| 176 | TGFB3 | na | 9555 | -0.639 | -0.3602 | Yes |
| 177 | ITGAV | na | 9564 | -0.640 | -0.3560 | Yes |
| 178 | PIK3R2 | na | 9597 | -0.645 | -0.3538 | Yes |
| 179 | EPAS1 | na | 9601 | -0.645 | -0.3492 | Yes |
| 180 | RET | na | 9656 | -0.655 | -0.3488 | Yes |
| 181 | COL4A1 | na | 9679 | -0.657 | -0.3457 | Yes |
| 182 | RASSF5 | na | 9758 | -0.667 | -0.3473 | Yes |
| 183 | COL4A2 | na | 9798 | -0.674 | -0.3455 | Yes |
| 184 | IGF1R | na | 9854 | -0.683 | -0.3450 | Yes |
| 185 | PRKCB | na | 9867 | -0.685 | -0.3408 | Yes |
| 186 | MSH6 | na | 9908 | -0.694 | -0.3390 | Yes |
| 187 | APC2 | na | 9933 | -0.699 | -0.3357 | Yes |
| 188 | HSP90AA1 | na | 10002 | -0.712 | -0.3361 | Yes |
| 189 | SMAD3 | na | 10010 | -0.714 | -0.3313 | Yes |
| 190 | E2F3 | na | 10031 | -0.717 | -0.3275 | Yes |
| 191 | TRAF2 | na | 10231 | -0.755 | -0.3387 | Yes |
| 192 | MTOR | na | 10269 | -0.763 | -0.3361 | Yes |
| 193 | MAPK3 | na | 10320 | -0.774 | -0.3345 | Yes |
| 194 | PIK3CB | na | 10335 | -0.777 | -0.3298 | Yes |
| 195 | COL4A4 | na | 10397 | -0.790 | -0.3290 | Yes |
| 196 | CCND1 | na | 10432 | -0.796 | -0.3258 | Yes |
| 197 | MMP9 | na | 10557 | -0.822 | -0.3301 | Yes |
| 198 | ITGA6 | na | 10612 | -0.836 | -0.3284 | Yes |
| 199 | APC | na | 10633 | -0.841 | -0.3237 | Yes |
| 200 | CTNNA2 | na | 10671 | -0.847 | -0.3204 | Yes |
| 201 | DVL1 | na | 10694 | -0.853 | -0.3158 | Yes |
| 202 | PLCG2 | na | 10697 | -0.853 | -0.3095 | Yes |
| 203 | PIK3CD | na | 10707 | -0.855 | -0.3038 | Yes |
| 204 | STK4 | na | 10730 | -0.861 | -0.2992 | Yes |
| 205 | MDM2 | na | 10838 | -0.889 | -0.3015 | Yes |
| 206 | FGF10 | na | 10866 | -0.897 | -0.2970 | Yes |
| 207 | PIK3CA | na | 10920 | -0.912 | -0.2946 | Yes |
| 208 | FADD | na | 10928 | -0.915 | -0.2883 | Yes |
| 209 | PIK3R5 | na | 10941 | -0.918 | -0.2823 | Yes |
| 210 | TRAF5 | na | 10958 | -0.925 | -0.2767 | Yes |
| 211 | TPR | na | 10973 | -0.929 | -0.2708 | Yes |
| 212 | PTCH1 | na | 10981 | -0.931 | -0.2644 | Yes |
| 213 | CBLB | na | 10996 | -0.935 | -0.2585 | Yes |
| 214 | KITLG | na | 10999 | -0.936 | -0.2515 | Yes |
| 215 | PIK3R3 | na | 11045 | -0.949 | -0.2482 | Yes |
| 216 | STAT3 | na | 11052 | -0.950 | -0.2415 | Yes |
| 217 | PIAS2 | na | 11080 | -0.959 | -0.2365 | Yes |
| 218 | TRAF6 | na | 11092 | -0.962 | -0.2301 | Yes |
| 219 | LAMA3 | na | 11104 | -0.965 | -0.2237 | Yes |
| 220 | BRCA2 | na | 11131 | -0.973 | -0.2186 | Yes |
| 221 | JAK1 | na | 11193 | -0.997 | -0.2162 | Yes |
| 222 | CDKN2A | na | 11228 | -1.009 | -0.2114 | Yes |
| 223 | LAMA1 | na | 11251 | -1.018 | -0.2056 | Yes |
| 224 | LAMA5 | na | 11261 | -1.022 | -0.1986 | Yes |
| 225 | PIK3R1 | na | 11265 | -1.023 | -0.1911 | Yes |
| 226 | MSH3 | na | 11318 | -1.044 | -0.1876 | Yes |
| 227 | ITGA2 | na | 11325 | -1.048 | -0.1802 | Yes |
| 228 | LAMB3 | na | 11340 | -1.052 | -0.1734 | Yes |
| 229 | TRAF3 | na | 11346 | -1.055 | -0.1658 | Yes |
| 230 | SOS2 | na | 11362 | -1.063 | -0.1590 | Yes |
| 231 | ETS1 | na | 11365 | -1.064 | -0.1511 | Yes |
| 232 | LEF1 | na | 11388 | -1.076 | -0.1449 | Yes |
| 233 | EGFR | na | 11415 | -1.085 | -0.1388 | Yes |
| 234 | GLI3 | na | 11456 | -1.100 | -0.1339 | Yes |
| 235 | DAPK1 | na | 11544 | -1.135 | -0.1327 | Yes |
| 236 | PIAS1 | na | 11577 | -1.157 | -0.1267 | Yes |
| 237 | FGF1 | na | 11593 | -1.171 | -0.1190 | Yes |
| 238 | APPL1 | na | 11675 | -1.222 | -0.1167 | Yes |
| 239 | BRAF | na | 11724 | -1.264 | -0.1112 | Yes |
| 240 | KIT | na | 11813 | -1.348 | -0.1084 | Yes |
| 241 | FAS | na | 11850 | -1.412 | -0.1008 | Yes |
| 242 | IGF1 | na | 11859 | -1.426 | -0.0907 | Yes |
| 243 | CBL | na | 11873 | -1.446 | -0.0808 | Yes |
| 244 | HHIP | na | 11914 | -1.539 | -0.0725 | Yes |
| 245 | FGF19 | na | 11936 | -1.633 | -0.0619 | Yes |
| 246 | FLT3 | na | 11939 | -1.644 | -0.0496 | Yes |
| 247 | RXRG | na | 12003 | -2.245 | -0.0380 | Yes |
| 248 | FGF13 | na | 12005 | -2.341 | -0.0203 | Yes |
| 249 | MAPK10 | na | 12008 | -2.724 | 0.0002 | Yes |
Table: GSEA details [plain text format]

  

Fig 2: KEGG\_PATHWAYS\_IN\_CANCER      
 Blue-Pink O' Gram in the Space of the Analyzed GeneSet

  

Fig 3: KEGG\_PATHWAYS\_IN\_CANCER: Random ES distribution      
 Gene set null distribution of ES for **KEGG\_PATHWAYS\_IN\_CANCER**
